# One-ecosystem analysis for environmental conservation and sustainable livelihood

**DOI:** 10.12688/f1000research.13999.1

**Published:** 2019-03-25

**Authors:** Helen T. Yap

**Affiliations:** 1 The Marine Science Institute, University of the Philippines, Diliman, 1101 Quezon City, Philippines

**Keywords:** Ecological research, environmental conservation, one ecosystem

## Abstract

In order to achieve the objectives of resource conservation, it is important to recognize that habitats are connected by material and energy flows, and that humans often play a central role, directly or indirectly. Hence, ecological research should be designed that treats all interlinked habitats, including human populations, as one ecosystem. Examples would be coastal habitats that are impacted by effluent from the upland, which in turn can be generated by deforestation and harmful agricultural practices. All of these aspects, including the river systems that deliver run-off, should be included in the investigation. This approach entails a carefully articulated hypothesis or set of hypotheses drawing on the natural as well as social sciences, and an appropriate sampling and statistical design. It clearly imposes high demands on resources, financial and otherwise. But the continued compartmentalization of efforts along disciplines and specializations will likely slow down progress in environmental conservation.

According to an existing platitude, all things are interconnected. However, this obvious fact does not necessarily underpin various forms of human endeavor. The focus of this commentary is on ecological research.

The tropics, whether terrestrial, freshwater, or marine, receive much deserved attention because they contain the highest concentrations of diversity on the planet. In marine scientific research, including studies on the coast, there is still a tendency to compartmentalize efforts, although the reasons behind this very likely have to do with pragmatic concerns such as funding, accessibility, and outright feasibility. Thus, initiatives still fall into categories such as coral reef ecology, integrated coastal management, mangrove forest research, seagrass investigations, and so on.

The habitats mentioned above are physically interconnected. An elegant study that illustrates this interconnection is that of McCauley *et al*. ^1^ at Palmyra Atoll, a remote collection of coral islets in the tropical central Pacific. In this ecosystem, the native forest plays host to seabird populations that indirectly contribute to nutrients in coastal waters through their guano, promoting the proliferation of zooplankton that in turn attracts feeding manta rays. Long trophic chains thus establish the unlikely connection between native trees and exotic marine creatures. The birds preferentially nest and roost in the more complex canopies of undisturbed forests and cause the elevation of nutrients in the underlying soils. The nutrient-enriched sediments then are washed off into adjacent coastal waters via precipitation and runoff. The ecological chains are readily disrupted by human activity, such as when the forests are replaced by commercial palm plantations.

This review promotes the viewpoint that since habitats (together with their associated human populations where applicable) are interconnected, they should not be studied as discrete entities but rather together as “one ecosystem”. For instance, a vast amount of literature in the past has emphasized the physical connectivity of coral reefs, seagrass beds, and mangroves ^2, 3^. This connectivity not only is enabled by the existence of a continuous medium (sea water) but is manifested in the exchange of energy and matter (dissolved and suspended compounds such as nutrients and sediments) and in the movements of organisms such as fish and invertebrates, mostly associated with particular developmental stages in their life cycles. Larvae typically are produced in particular “nursery” areas (seagrasses and mangroves) which offer the advantages of shelter and food. Later on, as juveniles, they migrate to other zones of the coast or the ocean to spend their adult phase foraging and reproducing. A study by McMahon *et al*. ^4^ used compound-specific stable isotope analysis to establish such a pattern for a fish species, the snapper *Lutjanus ehrenbergii*, as it used particular habitats in the Red Sea. Stable isotope analysis involves the use of amino acid δ ^13^C that is incorporated into the otolith (ear bone) of the fish from its diet. There are natural variations in this isotope, typically occurring at the base of food webs in different habitats. Otolith patterns revealed that the species spends its early life stages in coastal habitats such as seagrass beds and then migrates to offshore reefs where it lives and forages as an adult.

Some efforts in marine conservation, such as the establishment of marine protected areas, attempt to explicitly recognize these physical interconnections ^5^, although there are immediate implications in terms of the sizes of the areas to be covered, to be brought under some form of legal jurisdiction, and then to be monitored for implementation and compliance ^5^. All of these factors, in turn, affect pragmatic considerations such as investment in terms of financial support and human resources.

The reality of physical connectivity extends outside what is traditionally considered the marine realm or even the “coastal zone” ^6^. Studies have documented the physical link between the catchment in the upland and the coast, primarily through river runoff ^7, 8^. An example of a study that explicitly recognizes the need to integrate the investigation and understanding of habitats as a *single* ecosystem focuses on the Pacific island of Moorea, part of French Polynesia ^9^. The study is designed to enable experimentation on the entire ecosystem. Initiatives range from geology—mapping the topography of the rugged, mountainous terrain to high resolution—to detailed oceanographic studies, to taxonomic classification of all macroorganisms (greater than 1 mm in length) using DNA barcoding, all the way to socioeconomics such as the effects of tourism or of protection. The approach allows the generation of hypotheses and then the design of interventions—the “experiments”—at different spatial and temporal scales. A detailed compilation of past and current data will allow the formulation of mathematical models to predict the effects of such interventions, and empirical groundwork can compare these predictions with eventual outcomes. A recent study from Moorea ^10^ demonstrates that initiatives such as these continue to produce unexpected results; a case in point is found in the robust coral recruitment and recovery after an El Niño event (2016) that caused negative impacts elsewhere in the Pacific because of sea water warming.

The relevance of the one-ecosystem approach lies largely in the formulation and implementation of policy regarding land use or in managing the use of other types of natural resources. Humans are an integral part of the ecosystem; so any management plan should incorporate human welfare and quality of life as necessary goals ^11^. In addition, factors such as human attitudes and behavior can yield counterintuitive results if they are not adequately taken into account. Examples are attempts to regulate resource exploitation through government intervention such as by influencing market forces (in particular, market prices). This is illustrated in a recent case from the island nation of the Republic of Kiribati in the central Pacific ^12^. The population in Kiribati essentially divides economic activity between fishing and the cultivation of coconut for copra. Using standard economic theory, the government instituted a price increase in copra with the expectation that the pressure that fishing put on coral reef resources would be reduced. The results of this intervention, however, were compounded by characteristics of individual households, such as land ownership and possession of other economic assets. Overall, the price increase resulted in significantly increased labor devoted to copra but also to fishing. Households that owned the greatest amounts of land dedicated to coconut production exhibited the former trend whereas those with the smallest amounts of land for copra increased fishing the most, thereby negating the goal of coral reef conservation. This example highlights the need to include aspects of social science and even human psychology in ecological research ^11^.

One of the best demonstrations of the value of the one-ecosystem approach is the fact that rural households in the tropics typically switch among livelihoods because of weather conditions and the availability of resources ^13^. It is clearly unrealistic to categorize residents solely as “fishers” or “farmers”; members of even a single household can engage in each of these livelihood activities in addition to several others (teacher, government employee, tending a small retail store, and so on ^14^) depending on existing contingencies. The reality of multiple livelihoods makes it clear that habitats and resources cannot be assigned to compartments as is done in many routine investigations.

The one-ecosystem approach is not the same as the “ridge to reef” concept or even “ecosystem-based management” or “integrated ecosystem assessments”. “Ridge to reef” obviously refers to situations where coral reefs are at the receiving end of effluents from the catchment which could result in deleterious impacts downstream ^7^. The one-ecosystem perspective calls for considering the entire range of habitats and associated human populations together as a single entity, so that research and management interventions are designed accordingly from the outset (that is, the habitats and populations of interest together generate material and energy flows, defining the resulting natural processes). “Ecosystem-based” can still refer to just a single habitat (say, a coral reef or a seagrass bed), and “integrated ecosystem assessments” imply that separate habitats are still treated as separate ecosystems, and then subsequent data gathering and analysis are designed to capture the interfaces and interconnectedness (the process of “integration”) of these natural systems.

To reiterate, if coral reefs and seagrass beds, for example, are located along a coastline under investigation, they should be treated as a *single* unit *together with* any terrestrial habitats that exert a direct influence on them—via runoff, for instance—all the way to the highest pertinent reaches of the catchment. Treating the coral reef, seagrass bed, and the river conveying effluents up to the catchment as one ecosystem will dictate the type of data to be collected at relevant spatial and temporal scales. Obviously, there will always be a need for specific expertise on the biology and ecology of the range of organisms and associated communities encountered, including attendant physical, chemical, and geological processes, and on elements of the social sciences that are germane to the problem being investigated. Specialists should then work together to design the various indicators and parameters that ought to be measured to reflect the topology and complexity of the ecosystem. The operational boundaries of the latter will be determined on the basis of logistic considerations.

As is widely recognized, the one-ecosystem approach can entail costs that are prohibitive compared with those of more traditional, medium-term research investigations. Costs could be reduced by judicious selection of indicators on the basis of how effectively they would reflect responses to interventions, in terms of both natural processes and human behavioral change. This would be followed by careful and targeted assessment and measurement of these indicators, making use of proven scientific protocols. Efforts are best guided by what Bednarek *et al*. ^15^ call “boundary spanning” at the interface of science and policy. Research is specifically designed, and practitioners are purposely trained, to collect data and deliver evidence in a targeted manner to policy-makers and managers depending on specific, clearly defined issues and needs, usually centered on the goal of sustainability.

A useful demonstration of the effective connection between science and policy is a case study from Belize in Central America ^16^. This investigation covered a range of coastal habitats, including mangrove forests, seagrass beds, and an extensive coral reef. From the beginning, the focus was on ecosystem services derived from these natural systems, such as coastal protection, revenue from tourism, and a lucrative spiny lobster fishery. All habitats were treated as one integral whole, and there was continuous iteration between scientific planning and stakeholder input. Model scenarios informed the decision-making process, which resulted in a consensus favoring conservation mixed with development (while the other two scenarios represented extremes from pure conservation, all the way to unconstrained economic development of the coast). Future monitoring should test the robustness of model predictions which suggest a “win-win” solution combining conservation of critical habitats with sustained income from tourism and fisheries through selective development.

Obviously, in all of these efforts, a pervasive issue is that of climate change ^17^. The effects of increasing sea water temperature are demonstrated in dramatic fashion by the range expansion of benthic organisms such as reef corals ^18^. The overall trend for the tropics indicates that range expansion is accompanied by localized extinctions of species across numerous taxa and habitats—terrestrial, marine, and freshwater ^19^. The erstwhile tropics thus are being transformed into a kind of “hypertropics” as the typical tropical species vanish and move to higher altitudes and latitudes. Such fundamental ecological changes will certainly impact humans who traditionally depend on a number of these species for livelihood, whether wild-caught or cultivated. This brings us full circle to the need for a one-ecosystem approach as compelling global issues like climate change demand our attention. For analysis and understanding of natural systems, and eventual management of human activity to achieve sustainable livelihood, the one-ecosystem approach appears to be the only way to ensure success of conservation efforts.
